# Where Do We Go from Here? Prevalence of Trachoma Three Years after Stopping Mass Distribution of Antibiotics in the Regions of Kayes and Koulikoro, Mali

**DOI:** 10.1371/journal.pntd.0000734

**Published:** 2010-07-06

**Authors:** Sanoussi Bamani, Jonathan D. King, Mamadou Dembele, Famolo Coulibaly, Dieudonne Sankara, Yaya Kamissoko, Jim Ting, Lisa A. Rotondo, Paul M. Emerson

**Affiliations:** 1 Programme National de Lutte contre la Cécité du Mali, Bamako, Mali; 2 The Carter Center, Atlanta, Georgia, United States of America; 3 RTI International, Washington, District of Columbia, United States of America; 4 The Carter Center, Bamako, Mali; 5 International Trachoma Initiative, Decatur, Georgia, United States of America; University of Cambridge, United Kingdom

## Abstract

**Objectives:**

A national survey in 1997 demonstrated that trachoma was endemic in Mali. Interventions to control trachoma including mass drug administration (MDA) with azithromycin were launched in the regions of Kayes and Koulikoro in 2003. MDA was discontinued after three annual rounds in 2006, and an impact survey conducted. We resurveyed all districts in Kayes and Koulikoro in 2009 to reassess trachoma prevalence and determine intervention objectives for the future. In this paper we present findings from both the 2006 and 2009 surveys.

**Methods:**

Population-based cluster surveys were conducted in each of the nine districts in Koulikoro in 2006 and 2009, whilst in Kayes, four of seven districts in 2006 and all seven districts in 2009 were surveyed. Household members present were examined for clinical signs of trachoma.

**Results:**

Overall, 29,179 persons from 2,528 compounds, in 260 clusters were examined in 2006 and 32,918 from 7,533 households in 320 clusters in 2009. The prevalence of TF in children aged 1–9 years in Kayes and Koulikoro was 3.9% (95%CI 2.9–5.0%, range by district 1.2–5.4%) and 2.7% (95%CI 2.3–3.1%, range by district 0.1–5.0%) respectively in 2006. In 2009 TF prevalence was 7.26% (95%CI 6.2–8.2%, range by district 2.5–15.4%) in Kayes and 8.19% (95%CI 7.3–9.1%, range by district 1.7–17.2%) in Koulikoro among children of the same age group. TT in adults 15 years of age and older was 2.37% (95%CI 1.66–3.07%, range by district 0.30–3.54%) in 2006 and 1.37% (95%CI 1.02–1.72%, range by district 0.37–1.87%) in 2009 in Kayes and 1.75% (95%CI 1.31–2.23%, range by district 1.06–2.49%) in 2006 and 1.08% (95%CI 0.86–1.30%, range by district 0.34–1.78%) in 2009 in Koulikoro.

**Conclusions:**

Using WHO guidelines for decision making, four districts, Bafoulabe in Kayes Region; and Banamba, Kolokani and Koulikoro in Koulikoro Region, still meet criteria for district-wide implementation of the full SAFE strategy as TF in children exceeds 10%. A community-by-community approach to trachoma control may now be required in the other twelve districts. Trichiasis surgery provision remains a need in all districts and should be enhanced in six districts in Kayes and five in Koulikoro where the prevalence exceeded 1.0% in adults. Since 1997 great progress has been observed in the fight against blinding trachoma; however, greater effort is required to meet the elimination target of 2015.

## Author Summary

Trachoma, a blinding bacterial disease, is targeted for elimination by 2020. To achieve the elimination target, the World Health Organization (WHO) recommends member states implement the **SAFE** strategy; **s**urgery, mass administration of **a**ntibiotics, promotion of hygiene and **f**acial cleanliness and water and sanitation as **e**nvironmental improvements. We present results from evaluation surveys conducted in 2006 and 2009 from the regions of Kayes and Koulikoro, Mali. Prevalence of active trachoma in 2006 was below baseline intervention thresholds in all surveyed districts and the national program stopped antibiotic distribution. The prevalence of trachoma in 2009 remained well below levels in 1998. However, in 8 of 13 districts compared, the prevalence of active trachoma was higher in 2009 than 2006. Three years of antibiotic intervention did not equate in all districts to a sustained reduction of active trachoma. No surveillance activities were implemented after stopping interventions. Surgical interventions may have reduced the burden of blinding trachoma but there is an ongoing need for surgeries specifically targeting affected women. Four districts meet the WHO criteria for resuming district-wide mass antibiotic distribution. A community-by-community approach to elimination may be needed in other districts. The promotion of facial cleanliness and good hygiene behavior should be reintroduced.

## Introduction

Trachoma, a blinding bacterial disease of the conjunctiva, is targeted for elimination as a public health problem by the year 2020, yet an estimated 8.2 million people remain at immediate risk of blindness or visual impairment due to the disease [1]. To achieve the elimination target, the World Health Organization (WHO) recommends member states implement an integrated strategy of interventions known as **SAFE**: **s**urgery to correct trachomatous trichiasis; mass administration of **a**ntibiotics to treat current trachoma infections and reduce the infectious reservoir; promotion of hygiene and **f**acial cleanliness; and water and sanitation as **e**nvironmental improvements aimed at interrupting transmission of the infection. Based on WHO guidelines, districts are categorized for intervention based on the prevalence of clinical signs of disease: trachomatous inflammation follicular (TF) in children aged 1–9 years and trachomatous trichiasis (TT) in adults aged 15 years and older [2], [3].

Following a national trachoma prevalence survey in 1997, The National Blindness Prevention Program in Mali initiated a trachoma control program. Mapping of trachoma in Mali identified trachoma to be of public health significance throughout the country, including the regions of Kayes and Koulikoro where the prevalence of TF in children less than 10 years of age was 42.5% and 33.5% respectively [4]. The highest levels of TT among women 15 years of age and older were observed in Kayes (3.3%) and Koulikoro (3.9%) [4]. From 2002 to 2006 all sixteen districts in Kayes and Koulikoro received SAFE interventions to control trachoma. The interventions implemented in each region are listed in Table 1. Interventions were conducted in several ways: trained ophthalmic nurses moved from village to village offering free trichiasis surgery; mass distribution of oral azithromycin and tetracycline ophthalmic ointment occurred in annual campaigns for three consecutive years in each district following pilot distributions in target areas; facial hygiene, latrine construction and use, and the utilization of water for hygiene were promoted over local and regional radio stations; and persons in each region were trained to deliver health education and promote behavior change. The number of doses distributed and population coverage with azithromycin by district and year is shown in Table 2. In 2006, after three years of intervention and in accordance with the WHO guidelines, an impact evaluation was conducted to assess the effect of the SAFE activities [2]. The Ministry of Health withdrew A, and support for F and E interventions from partner organizations was limited. The Ministry of Health concentrated efforts to scale up the SAFE strategy in other regions yet to initiate interventions. The purpose of this study was to re-evaluate the prevalence of trachoma three years after SAFE interventions were discontinued in Kayes and Koulikoro. Here we present the data from the first impact evaluation in 2006 and the recent 2009 evaluation. We also aimed to quantify any need for additional interventions.

**Table 1:** SAFE Interventions in Kayes and Koulikoro Regions of Mali 2002–2006+.

	Kayes	Koulikoro
**S**urgery	15 Ophthalmic Technicians trained to perform trichiasis surgery3 371 persons operated*	10 Ophthalmic Technicians trained to perform trichiasis surgery4 651 persons operated*
**A**ntibiotic	Mass distribution of azithromycin3 079 437 doses distributedAverage coverage† of total populationRange by district 54.9–77.7%(see Table 2)	Mass distribution of azithromycin3 744 688 doses distributedAverage coverage† of total populationRange by district 43.9–86.1%(see Table 2)
**F**acial Hygiene	18 Local Non-Government Organizations trained in trachoma Information, Education and Communication (IEC)16 community radio stations commissioned to broadcast IEC messages	3 219 community IEC agents trained27 community radio stations commissioned to broadcast IEC messages
**E**nvironmental Improvements	81 wells were rehabilitated2 190 household latrines were constructed	75 community hygiene and sanitation committees were formed35 water point management committees were formed

**+:** Interventions are those performed by, or reported to the Programme National de Lutte contre la Cécité and do not include any other activities not targeted to trachoma control.

*: Cumulative number of persons operated to correct trichiasis from 2002–2008.

**†:** 3-year average coverage defined as: reported total number doses distributed divided by the census estimate for total population.

**Table 2:** District level antibiotic coverage of the total population in Kayes and Koulikoro Regions of Mali based on reported doses of azithromycin distributed† from 2002–2006.

District	2006 Estimated total population	Round 1 Doses distributed (% Coverage)	Round 2 Doses distributed (% Coverage)	Round 3 Doses distributed (% Coverage)	3-year average % coverage
**Kayes**					
1. Bafoulabé	205 228	93 542 (45.6)	42 823 (20.9)	201 749 (98.3)	54.9
2. Diéma	172 599	82 813 (48.0)	180 949 (104.8)	111 623 (64.7)	72.5
3. Kayes	390 223	165 933 (42.5)	327 820 (84.0)	158 421 (40.6)	55.7
4. Kéniéba	182 897	78 590 (43.0)	184 301 (100.8)	147 451 (80.6)	74.8
5. Kita	361 370	191 667(53.0)	86 556 (24.0)	330 646 (91.5)	56.2
6. Nioro du Sahel	201 551	94 213 (46.7)	204 907 (101.7)	170 783 (84.7)	77.7
7. Yelimane	147 735	59 629 (40.4)	142 058 (96.2)	84 704 (57.3)	64.6
**Koulikoro**					
8. Banamba	220 055	94 206 (42.8)	69 143 (31.4)	126 688 (57.6)	43.9
9. Dioila	212 977	139 255 (65.4)	120 735 (56.7)	226 582 (106.4)	76.2
10. Fana	199 487	150 640 (75.5)	118 401 (59.4)	119 205 (59.8)	64.9
11. Kangaba	90 928	65 680 (72.2)	70 385 (77.4)	98 787 (108.6)	86.1
12. Kati	611 471	273 605 (44.7)	365 552 (59.8)	341 688 (55.9)	53.5
13. Kolokani	215 270	76 105 (35.4)	174 880 (81.2)	90 694 (42.1)	52.9
14. Koulikoro	182 662	125 035 (68.5)	151 717 (83.1)	142 441 (78.0)	76.5
15. Nara	198 488	97 769 (49.3)	145 557 (73.3)	164 778 (83.0)	68.5
16. Ouelessebougou	169 185	62 022 (36.7)	115 935 (68.5)	109 352 (64.6)	56.6

**†:** Distributed is defined as the total number of doses reported distributed by the district health facilities during mass drug administration campaigns.

## Methods

### Ethical considerations

These prevalence surveys were conducted in accordance with WHO guidelines as part of the ongoing effort of the Ministry of Health to eliminate blinding trachoma in Mali and were necessary to evaluate the impact of interventions. In addition to the Ministry of Health, the survey protocol was approved by the Emory University IRB under protocol 079-2006. Informed verbal consent and assent was received according to the principles of the Declaration of Helsinki. Written consent was not obtained in these surveys due to the low literacy rate, ranging from 3% in rural Mali to 38% in Bamako (Enquête Démographique et de Santé 2001). Emory University IRB approved the use of informed verbal consent. Oral informed consent was sought first from village chiefs before surveys were conducted in the randomly selected villages. Consent was then obtained from household heads of randomly selected households and finally oral consent was obtained from each adult examined and consent from a parent or caretaker was obtained to examine children. Verbal assent was obtained also from children 6–14 years of age. Survey participants were informed of the purpose of the trachoma examinations and their rights not to participate or to stop the examination at any time. Choosing not to participate did not affect any decision in determining the need for interventions. Verbal consent was documented on a standard survey data collection tool. All children presenting signs of TF or trachomatous inflammation intense (TI) were offered free tetracycline eye ointment and instructed to apply it twice daily for 6 weeks. Persons identified with TT were recorded, counseled, and offered free consultation and surgery with a trained TT surgeon.

### Survey setting

**Figure 1:**
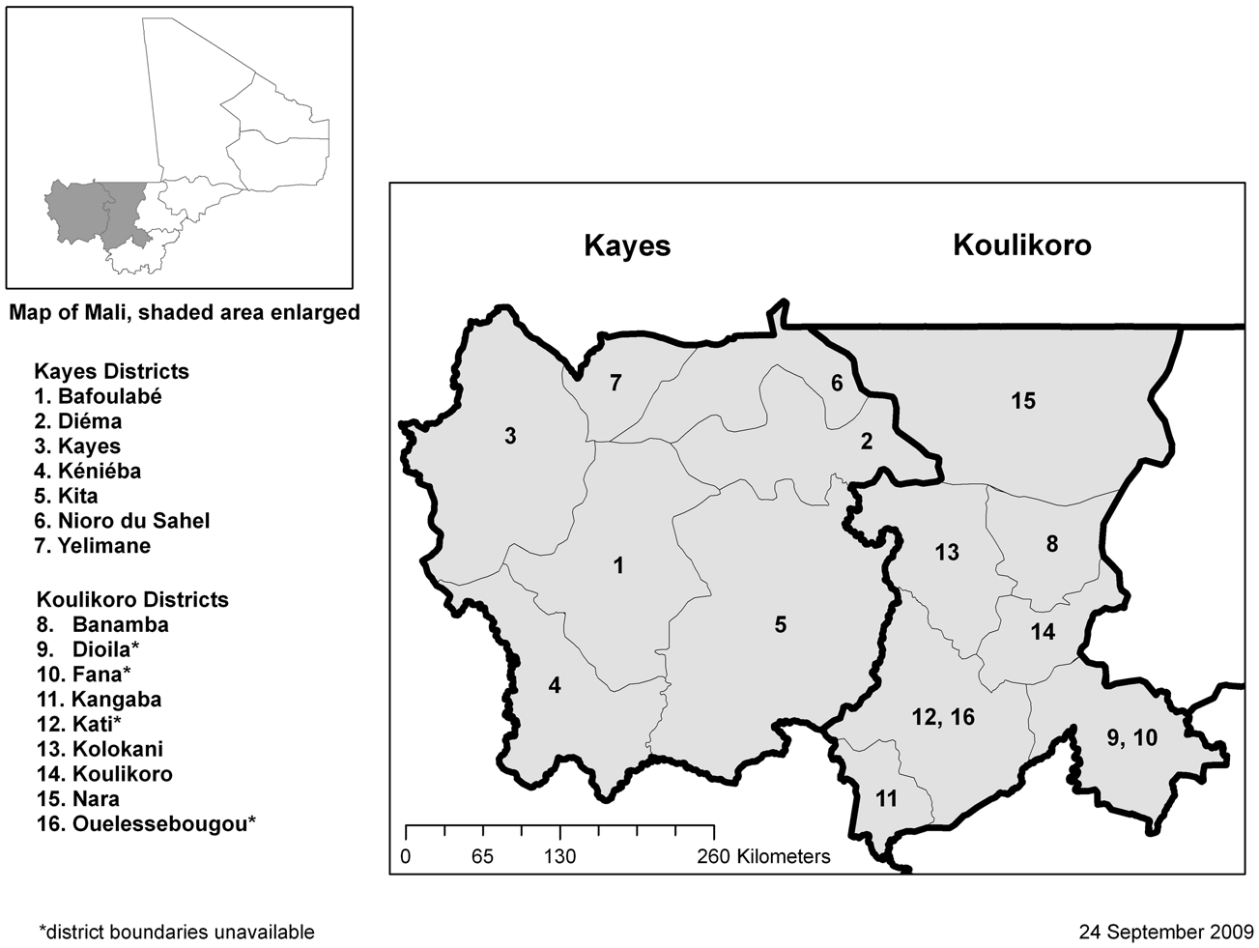
Regions of Kayes and Koulikoro, Mali.

Kayes Region is located in the extreme west of Mali bordering Mauritania to the north, Senegal to the west, and Guinea to the southwest (Figure 1). The region is divided into seven health districts with an estimated combined population of 1,763,987 persons (Mali National Demographic and Statistical Institute 2009 population projection). The primary ethnic groups are the Sarakole (Soninke) and Bambara.

Koulikoro Region is located in the western interior of Mali directly east of Kayes Region. It also borders Mauritania to the north and Guinea to the southwest. Koulikoro is divided into nine health districts with an estimated population of 2,072,185 persons (Mali National Demographic and Statistical Institute 2009 population projection). The primary ethnic groups are the Bambara and Malinke.

### Design/sampling

In both 2006 and 2009, population-based cross-sectional household surveys were conducted at the district level. Each survey in 2006 was done at least 6 months after the last round of antibiotic distribution following the implementation plan of the national program. Thus some districts in Kayes and Koulikoro were surveyed during the period between March and May and some during November and December. In 2009, all districts were surveyed during the period between March and May. Twenty villages (clusters) were selected from each district with a probability of selection proportional to the total population of the village. All villages with less than 5,000 total population from each district were eligible for selection. In 2006, 4 of 7 districts in Kayes (Bafoulabe, Diema, Kita and Nioro du Sahel) and all 9 districts in Koulikoro were assessed. In each of these districts, concessions (household compounds) were systematically selected using the random direction method [5]. All residents aged 1–9 years of age and 15 years of age and older from all households within selected concessions were examined for clinical signs of trachoma until approximately 60 qualifying children had been examined. In 2009, all 16 districts in the two regions were surveyed for a total of 320 clusters. Households within a cluster were randomly selected following the method of sketch mapping and segmentation which aimed to survey 24 households per cluster [6]. With the assistance of village leaders, survey teams drafted a list of all households, dividing households into segments of four. Village chiefs selected segments via lottery. All households in a selected segment were surveyed and all consenting persons over six months of age in each household were examined for trachoma. From the sampling methodology used in both surveys we assumed that the data was self-weighted.

### Clinical survey and questionnaire

Residents of selected households in 2009 were enumerated and designated as either present or absent. Absent was defined as not being physically present in the village on the day of the survey. Enumerated persons who were not at home, but in the village were found and recruited to the survey. Teams made at least one attempt on the same day to find residents marked absent during the first visit to the household. Absent residents in the 2006 survey were not enumerated. Clinical signs of trachoma were assessed using the WHO Simplified Grading System [3]. Examiners recorded the presence or absence of all trachoma grades in both eyes of survey participants using a ×2.5 binocular loupe and adequate light. The findings from the worst affected eye were reported. At examination in 2009, children were assessed for a clean face, defined as the absence of both ocular and nasal discharge. In 2009, each child 6–15 years of age was asked about their attendance in school, defined as public or private non-religious school. Attendance of Koranic schools or non-formal education was not assessed. Examined persons were asked about their participation in the most recent round of antibiotic distribution for trachoma control, defined as whether a person took oral azithromycin, applied tetracycline eye ointment, or did not participate. Estimates for participation in antibiotic distribution included only those persons present to give a response. Additionally in 2009, one adult respondent was interviewed in each household to determine the presence and use of a household latrine and the location of the main water source used by the household. The presence of a latrine was confirmed by direct observation and ‘use’ was defined as the observation of feces in the pit. The location of the water source was designated as within the household compound, within the village, or outside the geographical village boundaries as a proxy for distance and availability of water. Household interviews were not conducted in the 2006 surveys.

### Quality control and data management

Prior to the surveys, ophthalmic nurses were trained to use the WHO Simplified Trachoma Grading System through repetitive grading of digital photographs in a classroom setting and assessment of individual patients in the field. In 2009, these exercises were followed by a formal inter-observer reliability test of trachoma grading against a standardized set of 50 slides presented on a computer and a field exam of 50 children in which SB, DS and JDK were considered the reference examiners. Reference grading was supplemented with digital photographs. Eight out of ten ophthalmic nurses met the criteria of achieving greater than 80% reliability and a kappa statistic of 0.6 and above for grade TF and were selected as examiners for the survey. Survey teams were trained to randomly select households within a cluster, conduct household interviews, and record findings on standardized forms. A survey team consisted of one data recorder and one ophthalmic nurse. Formal inter-observer reliability tests were not conducted for the examiners in 2006.

Data were double-entered, compared and corrected. Based on the survey design used, we adjusted confidence intervals for the prevalence estimates and odds ratios to account for correlation among the data due to clustering using SAS SURVEY procedures (SAS version 9.2, SAS Institute Inc) [7], [8]. Regional prevalence estimates accounted for population differences between districts. We calculated differences between prevalence estimates from 2006 and 2009 and tested the equality of the estimates using the Z statistic with α = 0.05.

### Quantification of SAFE interventions

The ultimate intervention goal considered for achieving blinding trachoma elimination is the presence of less than 1 TT case per 1,000 population [9]. We calculated the total backlog of persons with TT in need of surgery by multiplying the 2009 point estimate and confidence limits of the population prevalence of TT by the estimated total population to give a point estimate and lower and upper bounds of the total number of people to be operated. According to WHO guidelines, where district-level prevalence of TF in 1–9 year-old children exceeds 10% at baseline, A, F and E activities are warranted district-wide and thus the total population living in these areas is targeted [2]. Where SAFE activities have been implemented, all areas that remain above 5% TF prevalence among children should continue antibiotic distribution [2]. The target prevalence by which mass antibiotic interventions to control trachoma is not needed is below 5% TF [2]. We calculated the number of household latrines required to achieve goal 7c of the United Nations Millennium Development Goals (MDGs); halve by 2015 the proportion of people who do not have access to improved sanitation [10].

## Results

### Surveys in 2006

In 2006, a total of 29,179 persons were examined from 29,779 persons available in 2 528 selected concessions. The mean number of concessions per district was 194.5 with a range by district of 110 to 312 concessions. A mean of 9.7 concessions were surveyed per village (range by village 1–25). The mean number of households per concession was 1.9 (range by concession 1–17). In the four surveyed districts of Kayes, 4,168 adults over 14 years of age and 4,808 children 1–9 years of age were examined for clinical signs of trachoma. In Koulikoro, 9,679 adults and 10,524 children were examined. Among examined adults over 14 years of age, 68.9% were women and among examined children 1–9 years of age, 51.5% were girls.

### Clinical observations

The prevalence estimates of clinical signs of trachoma in Kayes and Koulikoro in 2006 are presented in the first three columns by district in Table 3. Among adults 15 years of age and older, the prevalence of TT in Kayes was 2.37% (95%CI 1.66–3.07%, range by district 0.30–3.54%) and in Koulikoro, 1.75% (95%CI 1.31–2.23%, range by district 1.06–2.49%). The prevalence of trachomatous scaring (TS) among adults was 10.33% (95%CI 8.6–12.0%, range by district 3.0–18.4%) and 4.18% (95%CI 3.5–4.8%, range by district 0.6%–9.2%) in Kayes and Koulikoro respectively (data not shown). Prevalence of trachomatous corneal opacity (CO) in Kayes was 0.38% (95%CI 0.14–0.61, range by district 0.0–0.9%) and 0.31% (95%CI 0.12–0.51%, range by district 0.0–0.9%) in Koulikoro. Women were more likely than men to have TT (OR = 1.61, 95%CI 1.16–2.23, p = 0.004). Adults 50 years and older were more likely to have TT than adults aged 15–49 years (OR = 6.73, 95%CI 4.99–9.07, p = <0.0001).

**Table 3:** Prevalence of clinical signs of trachoma in Kayes and Koulikoro Regions, Mali 2006 and 2009.

	2006*			2009			
District	Adults ≥15 years	Children 1–9 years of age		All ages	Adults ≥15 years	Children 1–9 years of age	
	TT	TF	TI	TT	TT	TF	TI
**Kayes**	**n = 4,168**	**n = 4,808**		**N = 13,576**	**n = 6,566**	**n = 5,040**	
1. Bafoulabé	2.13 (1.1–3.1)	1.2 (0.1–2.4)	-	0.66 (0.29–1.04)	1.37 (0.6–2.1)	15.4 (11.7–19.0)	3.3 (1.7–4.8)
2. Diéma	2.53 (1.4–3.6)	5.4 (3.6–7.1)	1.8 (0.7–3.0)	0.91 (0.10–1.71)	1.87 (0.2–3.5)	4.7 (2.0–7.4)	1.3 (0.3–2.4)
3. Kayes	†	†	†	0.75 (0.42–1.08)	1.69 (0.9–2.5)	5.3 (3.4–7.2)	1.9 (0.9–3.0)
4. Kéniéba	†	†	†	0.78 (0.38–1.16)	1.65 (0.8–2.5)	7.1 (3.5–10.8)	0.3 (0.0–0.7)
5. Kita	3.54 (1.8–5.3)	5.2 (2.7–7.7)	1.6 (0.7–2.5)	0.74 (0.31–1.18)	1.52 (0.6–2.4)	2.5 (0.9–4.1)	0.4 (0.0–0.9)
6. Nioro du Sahel	0.30 (0.0–0.6)	2.8 (0.6–5.1)	0.3 (0.0–0.7)	0.59 (0.09–1.09)	1.26 (0.2–2.3)	8.7 (6.0–11.3)	0.5 (0.0–1.0)
7. Yelimane	†	†	†	0.20 (0.0–0.45)	0.37 (0.0–0.8)	6.5 (4.8–8.1)	2.9 (1.8–4.1)
Region	2.37 (1.7–3.1)	3.9 (2.9–5.0)	1.0 (0.6–1.5)	0.69 (0.53–0.85)	1.45 (1.1–1.8)	6.6 (5.7–7.5)	1.5 (1.1–1.8)
**Koulikoro**	**n = 9,679**	**n = 10,524**		**N = 19,342**	**n = 9,471**	**n = 7,139**	
8. Banamba	1.27 (0.3–2.2)	5.3 (3.5–7.0)	-	0.85 (0.48–1.23)	1.78 (1.0–2.5)	17.2 (13.7–20.7)	1.9 (0.9–2.8)
9. Dioila	1.53 (0.5–2.6)	0.6 (0.2–1.1)	0.2 (0.0–0.4)	0.67 (0.32–1.02)	1.25 (0.6–1.9)	7.9 (6.0–9.8)	1.0 (0.1–2.0)
10. Fana	1.32 (0.5–2.1)	2.3 (1.2–3.4)	0.1 (0.0–0.3)	0.19 (0.01–0.37)	0.34 (0.0–0.7)	3.9 (2.2–5.7)	0.9 (0.1–1.7)
11. Kangaba	1.25 (0.2–2.3)	2.9 (1.7–4.1)	2.0 (1.3–2.7)	0.31 (0.04–0.59)	0.68 (0.1–1.3)	2.1 (0.6–3.5)	0.7 (0.2–1.3)
12. Kati	2.49 (1.0–4.0)	2.3 (1.3–3.3)	0.4 (0.0–0.8)	0.57 (0.20–0.94)	1.07 (0.4–1.8)	8.4 (4.7–12.2)	0.3 (0.0–0.8)
13. Kolokani	2.05 (1.0–3.1)	5.6 (3.9–7.2)	0.7 (0.3–1.0)	0.76 (0.33–1.18)	1.55 (0.7–2.4)	14.1 (9.2–19.0)	0.3 (0.0–0.7)
14. Koulikoro	1.06 (0.2–2.1)	0.1 (0.0–0.3)	0.1 (0.0–0.3)	0.85 (0.41–1.29)	1.61 (0.7–2.5)	11.4 (9.1–13.7)	0.2 (0.0–0.5)
15. Nara	1.65 (0.9–2.4)	3.8 (2.0–5.6)	0.2 (0.0–0.6)	0.25 (0.0–0.54)	0.53 (0.0–1.2)	2.9 (1.5–4.2)	-
16. Ouelessebougou	1.09 (0.3–1.9)	1.3 (0.6–1.9)	0.1 (0.0–0.3)	0.26 (0.0–0.54)	0.55 (0.0–1.1)	1.7 (0.8–2.6)	0.3 (0.0–0.7)
Region	1.75 (1.3–2.2)	2.7 (2.3–3.1)	0.4 (0.2–0.5)	0.56 (0.43–0.69)	1.10 (0.8–1.4)	8.7 (7.5–9.9)	0.6 (0.4–0.8)

*Only persons aged under 10 years and 15 years and older were examined.

**†:** Kayes, Kéniéba and Yelimane districts were not surveyed in 2006.

- No cases were observed.

Parentheses show 95% confidence intervals.

District-level prevalence of TF among children 1–9 years of age had reduced to below the 10% intervention threshold in all surveyed districts. Among children 1–9 years of age, the prevalence of TF was 3.9% (95%CI 2.9–5.0%, range by district 1.2–5.4%) in Kayes and 2.7% (95%CI 2.3–3.1%, range by district 0.1–5.6%) in Koulikoro. The prevalence of trachomatous inflammation intense (TI) among children aged 1–9 years of age was 1.0% (95% CI 0.6–1.5%, range by district 0.3–1.8%) in Kayes and 0.4% (95% CI 0.2–0.5%, range by district 0–2.0%) in Koulikoro. Active trachoma (TF and/or TI) prevalence was 4.53% (95%CI 3.3–5.7%, range by district 1.2–6.5%) in Kayes and 2.96% (95%CI 2.5–3.4%, range by district 0.2–5.6%) in Koulikoro.

### Surveys in 2009

In 2009, from all districts in both regions a total of 42,128 persons were enumerated in 7,533 households and 32,918 were examined. In Kayes, a total of 13,576 persons were examined for signs of trachoma out of 17,127 persons enumerated from 3,287 households for a response rate of 79.3%. In Koulikoro, 19,342 persons were examined out of 25,001 persons enumerated from 4,246 households (a response rate of 77.4%). The response rate in women was 83.1% (17,771/21,386) and 73.0% (15,147/20,742) in men. The majority of adult men unable to be examined were absent from the home at the time of the household visit. Children 1–9 years of age composed 33.9% of the total enumerated population. Adults 15 years of age and older were 51.0% of the total population. The proportion of enumerated children 1–9 years of age who were examined was 88.2% and 77.1% of enumerated adults were examined. Among examined adults over 14 years of age, 58.1% were women and among examined children 1–9 years of age, 49.7% were girls. Among children 6–15 years of age the proportion that reported attending school was 42.6% in Kayes and 54.1% in Koulikoro.

### Clinical observations

The prevalence estimates of clinical signs of trachoma in Kayes and Koulikoro for 2009 are presented by district in the last four columns of Table 3. The prevalence of TT in the total population of Kayes region was 0.69% (95%CI 0.53–0.85%, range by district 0.20–0.91%). In Koulikoro, TT prevalence in the total population was 0.56% (95%CI 0.43–0.69%, range by district 0.25–0.85%). Among adults 15 years of age and older, the prevalence of TT in Kayes was 1.45% (95%CI 1.10–1.79%, range by district 0.37–1.87%) and in Koulikoro, 1.10% (95%CI 0.84–1.35%, range by district 0.34–1.78%). The prevalence of trachomatous scaring (TS) among adults was 4.22% (95%CI 3.7–4.8%, range by district 0.3–5.3%) and 4.68% (95%CI 4.1–5.2%, range by district 1.4–8.1%) in Kayes and Koulikoro, respectively (data not shown). Prevalence of CO in Kayes was 0.11% (95%CI 0.03–0.19, range by district 0–0.42%) and 0.21% (95%CI 0.13–0.29%, range by district 0–0.73%) in Koulikoro. Odds of TT among adults 50 years of age and older were ten times higher than adults 15–49 years of age (OR = 10.61, 95%CI 7.62–14.78, p<0.0001). Women were nearly two times more likely to have TT than men (OR = 1.85, 95%CI 1.40–2.46, p<0.0001).

At the regional level, the prevalence of TF was 6.6% (95%CI 5.7–7.5%, range by district 2.5–15.4%) among children 1–9 years of age in Kayes and 8.7% (95%CI 7.5–9.9%, range by district 1.7–17.2%) in Koulikoro. The prevalence of TI among children aged 1–9 years of age was 1.5% (95% CI 1.1–1.8%, range by district 0.3–3.3%) in Kayes and 0.6% (95% CI 0.4–0.8%, range by district 0–1.9%) in Koulikoro. Active trachoma (TF and/or TI) prevalence was 7.34% (95%CI 6.4–8.3%, range by district 2.7–16.8%) in Kayes and 8.91% (95%CI 7.7–10.1%, range by district 2.0–17.9%) in Koulikoro.

### Characteristics associated with uptake of SAFE strategy 2009

A total of 7,533 households were surveyed (range by district 423–480). The mean number of persons living in each household was 5.2 (SD = 2.7, range by district 4.5–5.9) in Kayes and 5.9 (SD = 3.0, range by district 4.9–6.9) in Koulikoro. Indicators of uptake of the A, F and E components of the SAFE strategy are listed by district in Table 4. The proportion of examined household residents reporting taking azithromycin or using tetracycline eye ointment in the most recent round of distribution was 86.1% (95%CI 84.2–88.0, range by district 54.6–99.8%) in Kayes and 83.9% (95%CI 81.6–86.3%, range by district 59.9–96.8%) in Koulikoro. Among children 1–9 years of age, 76.5% (95%CI 74.3–78.7%, range by district 46.7–95.2%) and 75.0% (95%CI 71.8–78.1%, range by district 52.1–86.8%) in Kayes and Koulikoro, respectively, had a clean face at examination. Basic sanitation (a household latrine) was evident in over 80% of the households in 12 out of the 16 districts. The presence of a latrine with evidence of use was observed in 88.1% (95% CI 85.2–91.1%, range by district 50.4–100%) of surveyed households in Kayes and 87.2% (95%CI 84.5–89.9%, range by district 37.4–99.8%) in Koulikoro. A water source inside the compound was observed in 9.7% (95%CI 5.5–13.9%, range by district 0.6–17.2%) of surveyed households in Kayes and 3.2% (95%CI 0.5–6.0%, range by district 0–11.8%) reported having to travel outside the geographical boundaries of the village to collect water. In Koulikoro, 19.8% (95%CI 15.6–24.0%, range by district 0–31.9%) of households had a source of water within the compound and 5.6% (95%CI 3.5–7.7%, range by district 0–17.2%) reported having to collect water from a source outside of village boundaries. Overall, there was no difference in the prevalence of clean faces between children living in households with water access inside the compound and children in households where the water source was outside the compound; 76.3% compared to 75.7%, Z = 0.48, p = 0.633.

**Table 4:** Indicators of A, F and E uptake in Kayes and Koulikoro Regions, Mali 2009.

District	%Residents taking antibiotics*	%Children 1–9 years of age with clean face	%Households (HH) with used latrine	%HH water source within compound	%HH water source outside village
**Kayes**					
1. Bafoulabé	99.8 (99.3–100)	95.2 (92.3–96.6)	100	10.0 (1.0–19.0)	4.4 (0.0–12.3)
2. Diéma	83.2 (81.6–84.8)	77.2 (63.8–77.4)	76.5 (61.3–91.7)	0.6 (0.0–1.6)	0.8 (0.0–2.6)
3. Kayes	-	94.7 (92.4–96.9)	100	8.3 (0.0–18.6)	5.0 (0.0–15.5)
4. Kéniéba	86.9 (84.9–88.9)	78.6 (73.3–84.0)	50.4 (31.8–69.1)	5.4 (0.4–10.4)	4.7 (0.0–11.3)
5. Kita	98.3 (96.6–99.9)	46.7 (39.3–54.1)	94.1(89.7–98.5)	17.0 (1.0–33.0)	0
6. Nioro du Sahel	54.6 (41.1–68.0)	62.5 (55.7–69.4)	78.2(66.7–89.8)	6.4 (0.0–14.4)	0
7. Yelimane	82.5 (80.6–84.3)	75.0 (70.4–79.6)	99.8 (99.4–100)	17.2 (7.5–26.9)	11.8 (3.9–14.3)
Region	86.1 (84.2–88.0)	76.5 (74.3–78.7)	88.1 (85.2–91.1)	9.7 (5.5–13.9)	3.2 (0.5–6.0)
**Koulikoro**					
8. Banamba	92.8 (90.6–95.0)	76.7 (70.8–82.6)	99.8 (99.4–100)	8.3 (0.6–16.1)	2.3 (0.0–7.1)
9. Dioila	93.4 (91.2–95.7)	74.5 (66.7–82.2)	94.8 (89.2–100)	15.5 (6.6–24.5)	11.8 (3.8–19.7)
10. Fana	96.8 (95.3–98.4)	81.6 (72.8–84.9)	97.3 (95.5–99.1)	19.1 (8.3–30.0)	17.2 (13.1–21.3)
11. Kangaba	59.9 (46.3–73.5)	52.1 (45.9–53.5)	86.3 (76.6–96.0)	31.9 (17.9–45.9)	0.4 (0.0–1.3)
12. Kati	78.0 (70.5–85.6)	75.0 (63.7–86.2)	87.6 (80.9–94.2)	30.1 (17.4–42.8)	3.7 (0.0–8.6)
13. Kolokani	92.2 (90.3–94.0)	76.3 (69.8–82.8)	86.6 (75.8–97.5)	10.2 (1.8–18.7)	0
14. Koulikoro	78.3 (66.8–89.7)	78.1 (74.7–81.4)	99.6 (98.9–100)	19.7 (9.9–29.6)	3.4 (0.0–8.7)
15. Nara	85.7 (83.5–87.9)	86.8 (83.0–90.6)	37.4 (21.9–53.0)	0	10.8 (0.0–23.8)
16. Ouelessebougou	65.4 (54.7–76.2)	57.7 (50.7–64.6)	89.5 (84.6–94.4)	28.8 (16.7–40.8)	0
Region	83.9 (81.6–86.3)	750 (71.8–78.1)	87.2 (84.5–89.9)	19.8 (15.6–24.0)	5.6 (3.5–7.7)

*Examined persons reporting a history of taking azithromycin or using tetracycline ophthalmic ointment during most recent round of mass distribution in 2005 or 2006.

- Data not available.

Parentheses show 95% confidence intervals.

### Difference between 2006 and 2009 estimates

**Figure 2:**
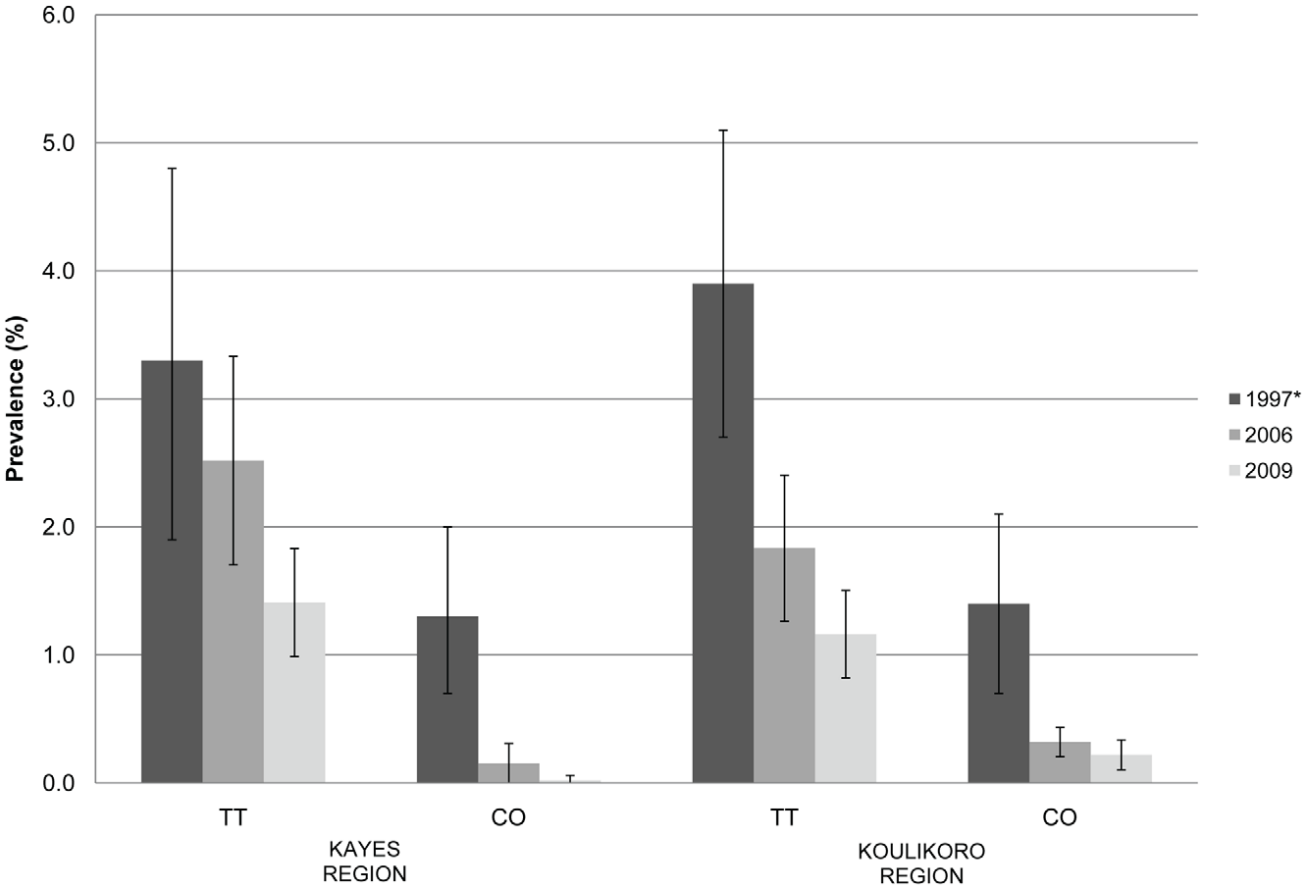
Prevalence of blinding trachoma (TT and CO) among women 15 years and older in Kayes and Koulikoro 1997*, 2006 and 2009. *regional estimate from Schémann et al 1998.

The regional estimates of prevalence of TT and CO among women 15 years of age and older from 2006 and 2009 are plotted in Figure 2 with the same estimates reported in 1997 for a comparison to baseline prevalence. In this age group, the difference in prevalence of TT between 2006 and 2009 for Kayes (Z = −1.33, p = 0.1829) and Koulikoro (Z = −1.78, p = 0.0744) regions was not statistically significant. There was no statistically significant difference in regional estimates of CO among adult women from 2006 to 2009 (Kayes Z = −1.01, p = 0.3117; Koulikoro Z = −0.44, p = 0.6626). However, among adults of both genders, the prevalence of TT in 2009 was less than the estimate in 2006 for both Kayes (Z = −2.06, p = 0.0396) and Koulikoro (Z = −2.79, p = 0.0052). The prevalence of CO among all adults between 2006 and 2009 did not differ in Kayes (Z = −1.21, p = 0.2245) or Koulikoro (Z = −0.55, p = 0.5838).

**Figure 3:**
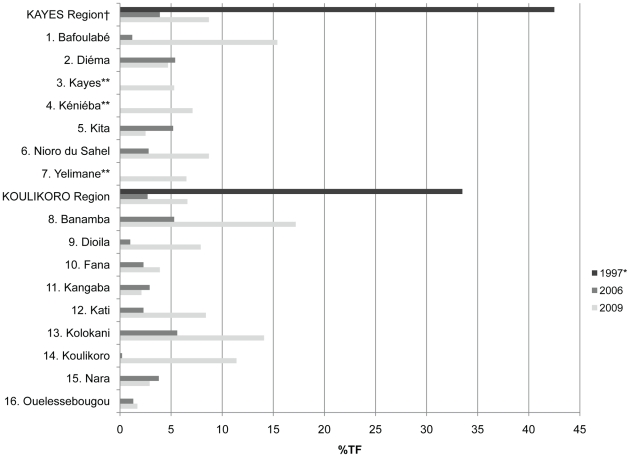
Prevalence of TF among children less than 10 years of age in Kayes and Koulikoro, Mali 1997*, 2006 and 2009. † estimate based on surveyed districts. *regional estimate from Schémann et al 1998. **districts not surveyed in 2006.

**Figure 4:**
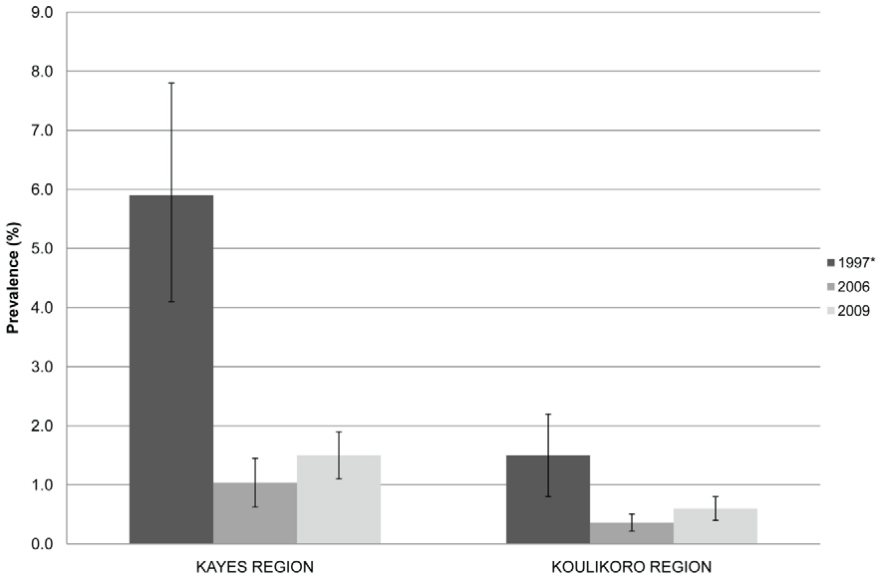
Prevalence of trachomatous inflammation intense (TI) among children under 10 years of age in Kayes and Koulikoro 1997*, 2006 and 2009. *regional estimate from Schémann et al 1998.

The regional prevalence of TF in 2009 was statistically greater than that observed in 2006 for both regions (Kayes Z = 8.13, p<0.0001; Koulikoro Z = 16.20, p<0.0001). The prevalence of TF for the region in 1997 is plotted with the district level estimates of TF from 2006 and 2009 in Figure 3. The differences in district level estimates between 2009 and 2006 with confidence intervals are listed in Table 5 along with Z statistic and p-values. The prevalence of TF observed in 2009 was statistically greater than that observed in 2006 for Bafoulabe, Nioro du Sahel, Banamba, Dioila, Fana, Kati, Kolokani and Koulikoro districts. The prevalence of TF in 2009 was the same or less than that observed in 2006 in the districts of Diema, Kita, Kangaba, Nara and Ouelessebougou. Regional estimates of TI among children from 1997, 2006 and 2009 are shown in Figure 4. Also for both regions, the prevalence of TI in this study was greater than that observed in 2006 (Kayes Z = 2.06, p = 0.0198; Koulikoro Z = 1.86, p = 0.0316). The prevalence of TI observed in 2009 was statistically greater than that observed in 2006 for Bafoulabe, Banamba, Dioila and Fana districts.

**Table 5:** Difference in district-level prevalence estimates of active trachoma signs among children 1–9 years of age in 2006 and 2009 in Kayes and Koulikoro Regions, Mali.

Outcome indicator	District	Prevalence change		
		2009–2006		
		Difference (95% CI)	Z value	P*
Trachomatous inflammation-follicular (TF) in children 1–9 years of age	1. Bafoulabé	14.1 (10.6, 17.7)	12.2	<0.0001
	2. Diéma	−0.6 (−3.9, 2.6)	−0.62	0.7323
	5. Kita	−2.7 (−5.6, −1.9)	−2.77	0.9972
	6. Nioro du Sahel	5.8 (3.0, 8.6)	5.37	<0.0001
	8. Banamba	12.0 (7.8, 16.2)	8.8	<0.0001
	9. Dioila	7.3 (5.5, 9.1)	8.2	<0.0001
	10. Fana	1.7 (−0.3, 3.6)	2.0	0.0205
	11. Kangaba	−0.8 (−2.6, 1.0)	−1.13	0.8698
	12. Kati	6.2 (2.3, 9.9)	6.12	<0.0001
	13. Kolokani	8.5 (3.9, 13.2)	7.00	<0.0001
	14. Koulikoro	11.3 (8.9–13.6)	10.62	<0.0001
	15. Nara	−0.9 (−3.4, 1.5)	−1.15	0.8756
	16. Ouelessebougou	0.4 (−0.7, 1.5)	0.77	0.2220
Trachomatous inflammation-intense (TI) in children 1–9 years of age	1. Bafoulabé	3.3 (2.1, 4.5)	6.35	<0.0001
	2. Diéma	−0.5 (−2.1, 1.1)	−0.83	0.7957
	5. Kita	−1.1 (−2.2, −0.1)	−2.18	0.9854
	6. Nioro du Sahel	0.2 (−0.5, 0.9)	0.65	0.2588
	8. Banamba	1.9 (1.0, 2.8)	4.72	<0.0001
	9. Dioila	0.9 (−0.2, 1.8)	2.45	0.0072
	10. Fana	0.8 (0.0, 1.7)	2.81	0.0025
	11. Kangaba	−1.2 (−2.2, −0.3)	−2.24	0.9874
	12. Kati	−0.1 (−0.8, 0.6)	−0.30	0.6186
	13. Kolokani	−0.4 (−0.9, 0.1)	−1.24	0.8930
	14. Koulikoro	0.1 (−0.3, 0.5)	0.61	0.2697
	15. Nara	−0.2 (−0.5, 0.0)	−1.43	0.9232
	16. Ouelessebougou	0.2 (−0.3, 0.6)	0.99	0.1616

*Probability that the difference in prevalence 2009–2006 is less than or equal to zero.

### Estimation of targets for continued SAFE interventions

Based on 2009 estimates, the total number of persons with TT who remain in need of surgery in Kayes is 10 967 (lower and upper bounds: 7,144 to 14,123) and 10,726 (bounds: 9,932 to 16,487) in Koulikoro. TT prevalence among adults exceeded 1% in 11 of 16 districts warranting continued, enhanced efforts to provide surgery to affected patients. While TT surgery may not be a priority in Yelimane, Fana, Kangaba, Nara and Oulessebougou where TT among adults is less than 1%, eye care facilities with the capacity to operate presenting TT cases should exist.

Mass distribution of antibiotics should resume in Bafoulabe, Banamba, Kolokani and Koulikoro where the prevalence of TF exceeds 10% among children. Additionally, according to WHO guidelines, mass distribution of antibiotics should continue in areas where after three years of intervention, the prevalence of TF remains greater than 5% among children under 10 years of age [2]. This would include communities within all of the 12 other districts of Kayes and Koulikoro. If this WHO guideline is interpreted at the district level, 10 districts in Kayes and Koulikoro regions warrant ongoing mass distribution of antibiotics, targeting a total population of approximately 2,637,492 persons.

The promotion of facial hygiene and environmental improvements should resume in all districts. Access to water within village boundaries and household latrine coverage was not lacking in most districts. Fana, Dioila, Yelimane and Nara had the highest proportion of households reporting having to collect water outside village boundaries. An estimated 11,526 households in Kayes and 19,718 households in Koulikoro must collect water outside village boundaries. The construction and maintenance of water points could be targeted to communities where access to water is lacking. Kenieba in Kayes and Nara in Koulikoro had the lowest proportion of households with a latrine. To ensure that every household has access to basic improved sanitation, 41,712 latrines need to be built in Kayes and 33,928 in Koulikoro. Building half of these by 2015 would meet MDG 7c.

## Discussion

The national survey conducted in 1997 providing baseline regional prevalence estimates were very useful in establishing the widespread nature of trachoma in Mali. In response to results from the national survey, trachoma control interventions were initiated in Kayes and Koulikoro Regions. Interventions were focused mostly on the S and A components of SAFE. F and E interventions were implemented, but had less geographical coverage of the target population than S and A. Until 2006, monitoring of interventions was limited to program reports and did not include rigorous field evaluations. According to reports of azithromycin distributed, antibiotic coverage was not consistent between districts or years with reported district level coverage ranging from 20.9% to 108.6% after the pilot phase in 2002. Several districts failed to reach the desired minimum of 80% coverage of total population in any one year and only one averaged above 80% over three years. These inconsistencies in coverage were either due to problems with the distribution or estimates of the target population. For example, the total population registered prior to MDA may have exceeded the census estimate of total population where coverage was greater than 100%. An evaluation of antibiotic distribution in Southern Sudan demonstrated the limitations of using distribution reports alone to calculate population coverage, as population estimates and treatment records can lead to inaccurate coverage estimates [11]. In Kayes and Koulikoro, we have defined “distributed” as the total number of doses reported to have been given out to individuals during mass drug administration campaigns, and caution that they have not been validated with coverage surveys. The figures are those reported by the district to the national program.

The first impact assessment in 2006 found prevalence of TF among children to be below 5% in 9 of 13 districts and below 10% in all districts. The programmatic decision was made to focus the available resources to other endemic regions that had not initiated SAFE interventions. This resulted in stopping mass antibiotic distribution and limited ongoing promotion of facial cleanliness and environmental improvements through schools and radio. Follow-up on the progress of latrine construction and new water points targeted for trachoma control stopped. Surgical services to correct trichiasis were maintained. Between the surveys in 2006 and 2009, it appears as though clinical signs of active trachoma returned in eight out of the thirteen districts.

The current data have several possible interpretations. There may have been a true decline in active trachoma from baseline to the present and this decline is associated with interventions from 2003–2006. Although prevalence of active trachoma signs are higher in some districts now than observed in 2006, the prevalence remains well below the 34% and 42% TF reported in Koulikoro and Kayes respectively during the 1997 baseline survey. National programs do not have control groups and it is not possible to determine whether the decline is due to the intervention, or to a secular decline, as has been described elsewhere [12], [13], [14], [15]. Prevalence of active trachoma has been observed to decline in the absence of a trachoma control program in the dessert region of Kidal [16]. We may also consider that there has been an heterogeneous effect of the interventions with some districts showing a sustained reduction in the prevalence of TF (Diema, Kita, Fana, Kangaba, Nara and Oulessebougou) and others showing a rapid rebound after initial control (Bafoulabe, Nioro du Sahel, Banamba, Dioila, Kati, Kolokani and Koulikoro). Such random effects are assumed possible by chance at the community level according to a stochastic model of trachoma transmission [17]. Models also suggest that trachoma endemicity at baseline is predictive of return of infection after antibiotic intervention [18], yet we have no district-level estimates at baseline on which to make assumptions. Antibiotic coverage is an important factor in the return of infection after treatment and thus the elimination of trachoma [17], [18], [19]. It is possible that high-risk marginalized sections of the population are systematically missed in mass drug administration leaving them untreated and able to repeatedly reintroduce infection into treated communities. Coverage surveys performed immediately following the mass distribution campaigns at least once during the three years of intervention may have identified any such problem.

Alternatively, there may be no difference between prevalence estimates of active trachoma in 2006 and 2009 due to the differences between the survey methods used and season of assessment in some districts, although this is unlikely given the scale of the observed differences and that seasonality of trachoma in Mali has not been established. In the 2006 survey, household selection methods may have biased the samples in villages where only a few large concessions were selected. The starting points were markets or mosques, structures typically at the center of a community and often surrounded by more populated concessions. Some clusters in the 2006 survey were composed of persons examined from households within a few, very large concessions, rather than the randomly distributed sample of households obtained using the sketch mapping and segmentation in the 2009 surveys. The household sampling in 2009 was more similar to that used in 1997 where a systematic random sample was taken from a listing of households within clusters. Both evaluations began with training ophthalmic nurses in the WHO simplified trachoma grading system. However, in 2009, the grader's reliability to diagnose TF was assessed rigorously and nurses not meeting a certain criteria were excluded from serving as a grader. This type of field assessment should improve the validity and reliability of a grader's findings.

The observed reduction in the prevalence of TT may have been a direct effect of the ongoing surgical services provided to TT patients. The diagnosis of TT is straightforward and allows less room for subjectivity than TF since the grade is based on one or more lashes touching the eye, rather than 5 or more follicles greater than 0.5 mm in diameter in the central part of the tarsal conjunctiva [3]. The grader's ability to identify TT is assessed in the classroom using slides but not in the field reliability assessment [2]. It may have been possible that graders under diagnosed TT in the field, but the possibility of this type of misclassification should not have differed from 2006 and 2009. Additionally, a greater proportion of adult males were absent from the household than females. TT impairs vision and thus compromises mobility; therefore men with healthy eyes may be more likely to be absent and not examined. Only if the reverse is true, men present in the household are more likely to have unhealthy eyes, would any bias in the prevalence of TT in men have masked any gender difference in TT. In both impact evaluations, women were more likely to have TT than men, which is consistent with findings from a recent review on the association of gender and trichiasis [20]. The statistically significant difference observed between TT prevalence among adults of both genders, but not among adult females from 2006 to 2009, may suggest a gender disparity in benefit from ongoing surgical services with men being more likely than women to present for surgery. Eliminating the backlog of trichiasis patients needing surgery remains a priority in both regions. Surgical services may need modification to specifically target women.

The indicators for A, F and E uptake (Table 4) obtained from the household surveys have several limitations. Although antibiotic coverage obtained from personal reports from household residents appears high, these results should be interpreted with caution. Residents were asked whether they had taken azithromycin during the most recent mass distribution campaign, which was in 2006. It is unlikely that residents could recall specifically taking drugs for trachoma given that mass drug distribution campaigns for other NTDs had occurred in more recent years. Additionally, only responses from residents available to respond were taken. These residents may have been more likely to have been available to receive antibiotics during campaigns than those residents absent from the household at the time of the survey, potentially inflating the coverage estimate. Not surprisingly, these personal reports are higher than coverage estimated by district distribution reports (Table 2).

More than 75% of households surveyed in each district, except Nara and Keneiba, had access to a household latrine with evidence of use. The evidence of use was determined by the presence of feces in the pit, which may be incorrectly interpreted as latrine use by all persons within the household. A latrine will be categorized as ‘in use’ if only a proportion of the household is using it. The role of latrines in reducing trachoma transmission assumes that where latrines are used, no open human feces is available for flies to utilize as a medium for egg and larval development; reducing successive fly populations and reducing the number of fly to eye contact. However, if use of a latrine is limited to only certain groups or if certain groups choose not to use the latrine, open defecation will continue. Further evaluation may be needed to assess actual behavior and potentially explain conflicting outcomes of endemic trachoma in the presence of high sanitation coverage as seen in Bafoulabe and Banamba districts. Assessing behavior is also necessary in determining the influence of water on trachoma. In this survey, there was no association of a clean face and having access to a water source within the boundaries of the household compound or having access to a water source outside of village boundaries. Additionally, greater than 80% of children were observed to have a clean face in only four districts; indicating that the practice of face-washing has not been fully accepted and adopted among residents in the two regions. On the contrary, our findings may also indicate that the ability of F and E components to control trachoma may not be as effective as anticipated. However, a recent analysis of factors associated with active trachoma in Mali supports the utility of face washing and environmental improvements [21].

One of the criteria for the certification of the elimination of trachoma is to demonstrate the sustained reduction of prevalence of TF among children below 5% for a period of three years after interventions have ceased [8]. In only six districts did the point estimate of the prevalence of TF remain below 5% at the district level from 2006 to 2009. In the 2009 survey the prevalence of TF among children was above 10% in four districts and above 5% in another six districts. With the recent global expansion of mass distribution of antibiotics for trachoma elimination, national programs may soon face a need to prioritize a limited quantity of drug [22]. Given such circumstances, Mali is facing unique programmatic decisions. Currently, WHO guidelines suggest the district be the implementation unit, but for certification of elimination, no community must have more than 5% TF among children [2], [9]. This suggests a community-by-community approach to trachoma elimination even in districts where district-level estimates of TF prevalence are below 5%. There are no recommendations or guidelines as to how a country such as Mali should attempt to demonstrate each and every community throughout the vast landscape has reached the elimination target. An acceptable level of TF prevalence at which the risk of developing blinding trachoma has been eliminated is unknown if the acceptable level is not zero. TF is not closely correlated with the presence of *Chlamydia trachomatis* DNA on ocular swabs and is thought to linger in the absence of infection [23], [24], [25], [26]. However, TI is better associated with DNA positive ocular swabs and is also linked to increased likelihood of progression to scarring, so is considered a more severe form of the disease [23], [27]. Ocular *Chlamydia* infection may have been significantly reduced by the interventions as evidenced by prevalence of TI in 9 districts of less than 1 child per 100. TI is more closely correlated to current infection with *Chlamydia trachomatis* than residual TF and has been suggested as a potential marker of infection post treatment [28]. In this setting, microbiological supporting evidence of the presence of bacteria would be useful, yet no guidelines exist for the use of laboratory diagnostics on a programmatic scale and it is perceived that costs of adding such tests to impact evaluations are prohibitive. Using TI for a proxy of infection, the prevalence of TI in 5 of the 10 districts in Kayes and Koulikoro qualified to receive mass distribution of antibiotics based on TF, indicates that less than 1 per 100 children would receive trachoma-specific benefits from the antibiotic.

Achieving less than 5% TF at the district level is achievable and can be feasibly determined on a programmatic scale through the cluster random survey design as demonstrated in this study and in Ghana [29]. Not considering the differences in survey methodology, a district level prevalence of less than 5% TF after three continuous years of heavy antibiotic intervention did not equate in all districts to a sustained reduction of TF below 5%. No surveillance activities were implemented after stopping AFE interventions in these districts. Doing so may have identified resurgence in districts with an apparent rebound in active trachoma and allowed immediate intervention. Results from these surveys provide evidence in the setting of a national program that antibiotics alone are not enough to eliminate trachoma. An analysis of associations between the components of the SAFE strategy demonstrates clearly that changes in hygiene behavior and improved sanitation can have protective effects against active trachoma [30], which argues for equal emphasis on hygiene and environmental improvements. Indicators used in the 2009 survey suggest very high access to sanitation in the two regions, but the indicators fail to capture actual behaviors. The promotion of facial cleanliness and good hygiene behavior should be reintroduced in all districts of Kayes and Koulikoro. Surgical services to correct trichiasis should also be continued, but where and for how long to continue mass distribution of antibiotics is not as clear. Currently, the 4 districts with TF above 10% among children are priority for mass distribution of antibiotics. More guidelines from the international community are urgently required to help prioritize the limited quantity of donated antibiotic in addition to recommending appropriate evaluation methodology for determining when certification targets have been achieved.

## Supporting Information

Checklist S1 STROBE checklist
(0.08 MB DOC)
